# The psychiatric interview: validity, structure, and subjectivity

**DOI:** 10.1007/s00406-012-0366-z

**Published:** 2012-09-23

**Authors:** Julie Nordgaard, Louis A. Sass, Josef Parnas

**Affiliations:** 1 Psychiatric Center Hvidovre, University of Copenhagen, Broendbyostervej 160, 2605 Broendby, Denmark; 2 GSAPP-Rutgers University, 152 Fredlinghuysen Road, Piscataway, NJ 08854 USA; 3 Psychiatric Center Hvidovre and Center for Subjectivity Research, University of Copenhagen, Njalsgade 140-142, 2300 Copenhagen, Denmark

**Keywords:** Consciousness, Epistemology, Sign, Psychiatric interview, Subjectivity, Symptom

## Abstract

There is a glaring gap in the psychiatric literature concerning the *nature* of psychiatric symptoms and signs, and a corresponding lack of epistemological discussion of psycho-diagnostic interviewing. Contemporary clinical neuroscience heavily relies on the use of fully structured interviews that are historically rooted in logical positivism and behaviorism. These theoretical approaches marked decisively the so-called “operational revolution in psychiatry” leading to the creation of DSM-III. This paper attempts to examine the theoretical assumptions that underlie the use of a fully structured psychiatric interview. We address the ontological status of pathological experience, the notions of symptom, sign, prototype and Gestalt, and the necessary second-person processes which are involved in converting the patient’s experience (originally lived in the first-person perspective) into an “objective” (third person), actionable format, used for classification, treatment, and research. Our central thesis is that psychiatry targets the phenomena of consciousness, which, unlike somatic symptoms and signs, cannot be grasped on the analogy with material thing-like objects. We claim that in order to perform faithful distinctions in this particular domain, we need a more adequate approach, that is, an approach that is guided by phenomenologically informed considerations. Our theoretical discussion draws upon clinical examples derived from structured and semi-structured interviews. We conclude that fully structured interview is neither theoretically adequate nor practically valid in obtaining psycho-diagnostic information. Failure to address these basic issues may have contributed to the current state of malaise in the study of psychopathology.

## Introduction

Highly structured interviews have become the gold standard of diagnostic interviewing in psychiatry, primarily in research but also, increasingly, in ordinary clinical work. The literature on psychiatric interviewing usually deals with comparisons of the relative efficacy (degrees of sensitivity and specificity) and reliability of particular interview approaches. Typically, these discussions fail to address the more overarching theoretical issue, namely: What is the epistemologically adequate manner of obtaining psycho-diagnostic information? Even the textbooks and chapters devoted to psychiatric interview tend to be mute on the theoretical underpinnings of interviewing [2]. We have not, in fact, found in the literature any single contribution that systematically addresses ontological and epistemological foundations (“ontological” refers here to the nature of being of psychiatric symptom and sign) of the psychiatric interview (although a recent paper by Stanghellini [67] comes close). In other words, there seems to be an important lacuna in the psychiatric literature concerning the interview-relevant basic concepts on the nature of symptom and sign (what Berrios calls the “psychiatric object” [4, 42] and the methods used to elicit and describe them. The task of this paper is to address this lack in a way that is theoretically coherent and reflects practical clinical reality.

The goal of a psychiatric assessment is to describe the patient’s complaints, appearance, and existence in an actionable psychopathological format, namely, one that results in diagnostic classification and other clinical decisions. This process includes, to a large degree, describing the patient’s experiences, originally lived in the first-person perspective, in potentially third-person terms, thus providing “objective” data that can be shared for diagnosis, treatment, and research. We exclude from consideration “a free-style clinical interview” which grants the clinician total liberty, thus failing to prevent limited comprehensiveness (due to lack of systematic exploration of psychopathology) or guard against incompetence. This type of interview has been shown to be notoriously unreliable [13].

For the sake of illustration, we will articulate, as a part of our presentation, a contrast between two types of interviewing:

1. a fully structured psychiatric interview, performed by a clinician psychiatrist or psychologist or even a non-clinician (a student, a nurse, etc.) who has been specifically trained for this purpose. This sort of structured interview consists of asking the patient pre-specified questions in fixed sequence and rating the responses as positive, negative, or threshold [16]. The fully structured interview relies on a series of assumptions that we wish to explore.
2. a conversational, phenomenologically oriented, semi-structured interview, performed by an experienced and reliability-trained psychiatrist. The “structured” component in the “semi-structured interview” consists of a list of items (typically, an aggregate of relevant scales) on which the interviewer *must* elicit sufficient information in order to score these items after completing interview session. Here, however, the flow of the interview is conversational. Questions are contextually adapted and follow the train of the patient’s narrative, yet with a constant opportunity to ask for more detail or further examples (this includes the possibility of a gentle interrupting and changing the direction of the interview). Spontaneity, recollection, and reflection on the part of the patient are strongly encouraged. “Yes/no” answers never suffice but always require exemplifications in the patient’s own words. The specifically phenomenological aspects will be articulated in the course of the paper, but briefly put; this approach aims at a faithful recreation of the patient’s subjective experience.

The issue at hand touches upon many topics from the philosophy of science, philosophy of mind (viz., consciousness, its description, psychophysical relation, etc.), cognitive neuroscience, semantics and semiotics (theory of meaning), linguistics, anthropology, and affective science. An exhaustive, scholarly review of all these issues is obviously beyond our scope. We restrict ourselves to a few indispensable and pragmatically relevant aspects of clinical phenomenology and philosophy of mind.

## The origins of structured interview

The development of the structured interview was prompted by the need for improving reliability of psychiatric assessments. As is well known, the WHO-sponsored US–UK diagnostic project [9] demonstrated markedly different diagnostic habits of British and American clinicians. It was clear from these studies that *a science of psychiatry* was not possible without strengthening the reliability of psychiatric assessments. The project also demonstrated that the diagnostic differences could be minimized by using a standardized structured interview and shared diagnostic criteria [9].

The US–UK study served as an important impetus for the “operational revolution,” leading to criteria-based diagnoses, “operational” definitions of such criteria, and a strong emphasis on interrater agreement, a development vigorously spearheaded by an influential psychiatric group from Washington University in St Louis, Missouri (the so-called Neo-Kraepelinian movement). The New York Post—in a very enthusiastic tone—described these first attempts as “a new tool that rolled psychiatrist’s thermometer, microscope and X-ray machine into one” (quoted in [66]). The criteria of diagnostic categories, eventually summarized in the DSM-III+ and ICD-10, became, with the passage of time, the catalog of officially sanctioned symptoms and signs, while the remaining psychopathological features largely went into oblivion [1] and are no longer mentioned in the major textbooks. The interview schedules are constructed to be as directly compatible with the diagnostic criteria as possible, to the point that the criteria are often used as the interview questions.

Robert Spitzer, an important figure behind the DSM-III+ project, justified the creation and use of the structured psychiatric interviews in his famous paper: “Are clinicians still necessary?” [66]. Interviews, he argued, could be more reliably performed by economically affordable, naïve raters who would stick to the pre-formed questions than by clinicians who were both expensive and unreliable. It is important to emphasize that during these transformative years for psychiatry, improved interrater reliability was just about the only *conceptual or clinical argument* offered to justify the operational project. It needs also to be noted that the notion of operational definition [6, 25], defined as a set of rules whose execution links the concept with its referent in empirical reality in a clear-cut, unambiguous way (e.g., “ice = volume of water that changes into solid state when temperature drops below zero Celsius”) is inapplicable to psychiatric concepts, as emphasized by Manfred Spitzer [65] (see also [52]). In psychiatry, we typically do not, and cannot, operate with concepts that are operationalizable in the above sense. The recognition of a depressive state, inappropriate affect, or a paranoid style, for instance, cannot readily be associated with any easily identifiable, observable facts. Or consider such current DSM criteria as “identity disturbance … with unstable self-image or sense of self” or “bizarre delusions,” “depressed mood” or feeling “restless… or keyed up or on edge,” or even “a pattern of unstable and intense interpersonal relationships.” (re: borderline personality disorder, schizophrenia, dysthymia, generalized anxiety disorder). All these features require forms of judgment and complex pattern recognition that challenge or defy Bridgman and Hempel’s conceptions of operationalizing. In general, the psychometric concepts used in psychiatry stem from psychometric theory in psychology but without serious considerations about their relevance and applicability to the psychiatry [3, 28].

During these transformative years for psychiatry, behaviorism was a dominant force in psychology [64]. Consciousness, experience, and notions like “the self” were epistemologically “incorrect” terms, banned and relegated (with the brain) into the domain of “the black box,” with the focus put rather on studying *input and output* correlations (stimulus–response paradigm) in a setting of operant conditioning. In sum, psychiatry did not possess adequate conceptual resources to deploy when it was redesigning its classificatory principles and nosological categories. And psychiatry continued to apply behaviorist or operationalist approaches even after these were abandoned in psychology (on its way to becoming cognitive neuroscience) and the philosophy of science, where more complex notions of the scientific enterprise were taking hold [52]. In psychiatry, there was virtually no discussion of such crucial issues as the nature of consciousness and its study, or the status of first-, second-, and third-person perspectives. Even quite recent publications do not provide any theoretical or empirical-phenomenological arguments in favor of the structured interview.

Historically speaking, the most comprehensive analysis of psychiatry’s theoretical foundations was offered in successive editions from 1913 to 1954 by a German psychiatrist and philosopher, Karl Jaspers. Despite an English translation in 1963, the text had limited impact on psychiatric practice and research in the Anglophone world. Many of our key points in this article are anticipated in Jaspers’ book ([31, 32]), as Jaspers himself based many of his insights from the emerging science of the humanities [12, 73]. His vision of psychopathology placed a decisive emphasis on phenomenology, in the sense of a systematic exploration of the patient’s subjective experience and point of view. The object of psychopathology was the “conscious psychic event,” and psychopathology consequently requires an in-depth study of experience and subjectivity.

## Structured interview and phenomenology: a case vignette

The following case vignette is selected from our series of ongoing studies of first-admission patients who show possible signs of beginning psychotic illness [21, 54]. The patient is a 27-year-old, unmarried man, with a high-school education, who felt different from other people and, ever since childhood, sensed that no one understood him. He had no friends, isolated himself, and avoided contact with others. He devotes his time to various kinds of art, mostly painting, and takes an art class now and then. At 19, he suffered from a brief episode in which he suddenly stopped speaking, heard voices, and had very aggressive, violent thoughts; this lead to hospitalization for a few days.

Table 1 juxtaposes the patient’s responses to thematically corresponding questions in A. a, structured interview and B, a conversational, phenomenologically oriented semi-structured interview performed on another occasion. A number of typical, theoretical problems are well illustrated by the responses yielded in these distinct interviewing contexts.

**Table 1:** Example of different information elicited in the different approaches

Structured interview	Conversational interview
He has always felt depressed and it gets worse from time to time. For the last month, he felt stressed, sad, and angry	He feels depressed, angry, and anxious; and notes as well that he has changed the last couple of years, for example, he finds it “very strange to look out through these two eyes”
Diminished interest and loss of joy	Often, he feels “caught by sadness and racing thoughts,” it all began when he was 6 years old
Insomnia, tiredness, and lack of energy	Awake during the night and sleeps during the day. When awake, he paints, reads, and plays computer
Difficulties with thinking and concentration	Some problems with concentration, but none when engaged in artwork or computer games. Yet he hears his thoughts aloud in his head
Denies delusions	Experiences alien thoughts being inserted into his head as a “bad breath”
	He doubts his own existence and often looks in the mirror to check whether he exists
Sometimes he feels that he is “the center of attention,” but knows that he is not	He feels that people are starring and laughing at him
	Always felt that he “lives in his head,” his body is just a vehicle
Thoughts about death and suicide	Suicidal thoughts and thoughts about “doing an experiment,” for example, to strangle a dog or stab his mother with a knife, “just to see what would happen.” These thoughts seem alien to him

The structured approach yields a diagnosis of major depression. The positive “depressive responses” take on a rather different cast, however, in the clinical-phenomenological exploration; indeed they seem to change identity. For example, the affirmative answer to the suicidal-ideation question in the structured interview, takes another turn in the phenomenological approach, with indication of bizarre aggressive ideation, experienced with a certain sense of passivity. Also revealed is a reversal of diurnal rhythm, suspicion of influence phenomena (thought insertion), and various self-disorders (disorder of first-person perspective, disembodiment, diminished self-presence, diminished sense of basic identity) [26, 49, 53]—all suggestive of a schizophrenia-spectrum disorder.

Two sorts of problems are apparent here. One pertains to fairly straightforward information that was not asked about (e.g., diurnal rhythms); the other more subtle issues that often require a different *kind* of dialog (e.g., self-disorders). Both problems are related to what artificial intelligence researchers in cognitive science call the “frame problem,” the issue of how to decide what is relevant, indeed what is even the relevant overall *context* within which to approach a given problem [62]. The very nature of the structured interview precludes the sorts of relevance judgments and frame shifting that cognitive research shows to be necessary in situations requiring complex pattern recognition, which is obviously the case in most psychiatric interviews.

Difference in the process of eliciting information is illustrated in the following transcripts, first from a structured and then from a conversational, phenomenological interview with the same patient. Table 2 shows questions and answers from a structured interview and Table 3 questions and answers from a conversational, phenomenologically oriented interview.

**Table 2:** Transcript from a structured interview

Interviewer	Patient
Have you ever experienced that certain thoughts that were not your own were put into your head?	Hmm, no
What about thoughts being taken out of your head?	Shakes his head to deny

**Table 3:** Transcript from a conversational, phenomenologically oriented interview

Interviewer	Patient
Does it ever become too much with all these thoughts? (referring to previously addressed experience of difficulties in concentration and of thought pressure)	Sometimes, I think the thoughts take somehow over, so I cannot get rid of them. Then the thoughts run their own race
Can you say some more about that?	It’s like the thoughts are out of my control
Can you get a feeling that the thoughts are somehow alien… or not really your thoughts?	Yes, sometimes it is like they are… when the thoughts are kind of solemn thoughts or, how to put it, then I can get the feeling that they have been sent from another place, from elsewhere. Because, if they are not mine, and they are solemn thoughts, then they must be something special
Do you have any idea from where they could have been sent?	From God
What are solemn thoughts?	They are very different from my usual thoughts and are thoughts that other people don’t think
And then you think that God is sending you these thoughts?	Yes

It seems the patient actually *does* experience thoughts being inserted into his head. Why then did he not reveal this in the structured interview? One reason might be that he does not recognize his own experience in the rather blunt, implicitly either/or formulation of the structured-interview question. Another possibility is that the experience of insertion does not fully articulate itself for the patient until he starts to talk, in more general terms, of his experience of more subtle, albeit disturbing, alterations of the stream of consciousness (with its apparent progression through concentration difficulty to thought pressure, to thoughts acquiring autonomy, to alien thoughts, to thought insertion, and on to delusional explanation).

A characteristic feature of the structured interview is the danger of over confidence in the face value of the answers, as if a simple “yes” or “no” truly confirmed or denied the diagnostic criterion at issue. There is an implicit assumption that symptoms exist as ready made, *pre*-*defined mental objects, waiting* *in the patient’s (access) consciousness* for an adequate prompting to come into a full view. To put it in another way, the structured interview *pre*-*defines what counts as information*. The nature of this information is conceived on analogy of a substantial, temporally enduring thing, almost like a table or a chair. Having glimpsed the potential problems at the concrete clinical levels, we will now look more systematically at the theoretical assumptions that seem implicit in the (overtly atheoretical) psychiatric literature on interviewing.

## The process of typification

Philosophical and cognitive-scientific (e.g., [56, 57]) analysis of categorization, together with empirical studies of the diagnostic process itself [35] offers a convergent picture of the actual, real-world process of coming up with a psychiatric diagnosis. They show that, in most cases, the information provided by the patient coupled with his behavior, experience, and psychosocial history leads, in a natural conversational clinical situation, to the first typifications, that is, to the interviewer’s *seeing the patient as resembling a certain prototype* [59, 60].

In a diagnostic encounter, for example, the psychiatrist quickly senses a patient as being in *a certain way*, for example, as withdrawn, hostile, sympathetic, guarded, eccentric, etc. As the interview progresses, these initial typifications become more specific and nuanced, modified by further interactions with the patient [60]. Initial typifications evolve and may sometimes be replaced; but there is always some typification that functions as a formative matrix upon which specific features and responses are assessed. The concept of typification refers to a very basic human cognitive feature, especially pertinent in perception, namely that perception of an object is always *apperceptively organized*, that is, structured, in a semi-conceptual fashion, as a salient unity or a certain Gestalt. In this sense, one might say, seeing is always “seeing as …” [22, 46, 75]; it is always perspectival or aspectual. It involves pattern recognition and pattern completion, thereby allowing apprehension of objects and situations under conditions of limited or incomplete information. Typification is a largely automatic process that pervades all of our experiences and occurs outside explicit awareness. In typification, an interpretation is not superimposed on a perceptual act; it imbues the perception itself. At least in the normal sense, for example, we do not recognize a face as friendly based on logical inference from perceptions of individual muscular contractions on the other person’s face; we see it directly “as friendly.”

Obviously there are potential dangers in typifications; first, that the psychiatrist can be blinded by his or her expectations and therefore may fail to recognize subsequent data for what they really mean. Second, the repertoire of typifications that any psychiatrist has acquired through past experience could always contain various misperceptions and misconstruals. Third, typifications could be misused as stereotypes if the clinical investigation does not advance toward a gradually more individualized understanding of the patient.

Typification is, however, a fundamental and indispensible constituent of the diagnostic process [60] and a way to give order and meaning to psychic states by revealing the ideally typical connections instead of a disjointed enumeration of them [31, 32]. The scientific use of typifications requires that psychiatrists also doubt and reflect on their typifications, and repeatedly test their own interpretations by looking for additional components to prove their typification or call it into question. Typifications are scientific only to the extent that they are based upon and tested by evidence, given through direct observation and communication with the patient. The value lies in orienting and structuring the first steps in psychiatric investigations [60].

The process of typification, crucial in the phenomenological approach, is, however, eliminated or severely constrained in a fully structured interview, in which both the relevant expressions (observable signs) and “inner” experiences (symptoms) are selected in an a priori fashion, in the sense of being, assumed to be pre-defined as well-demarcated, mutually independent entities. Let us now look more closely at some of the epistemological issues involved.

## The dichotomy of “inner and outer” and the notion of Gestalt

The term “symptoms” refers, of course, to the patient’s subjective complaints whereas “signs” refers to third-person phenomena that are “externally” observable. This distinction is mainly unproblematic in somatic illness, where symptoms and signs share their ontological, thing-like, nature (see below), ideally pointing to their somatic causes. By contrast, the vast majority of psychiatric *signs* are *expressive*, linked to emotion, mood, interpersonal rapport, bodily movement and (pre-eminently) language, and discourse—all of which involve a subjective component. We can of course artificially separate the expression from the expressed content when scoring a mental status examination (e.g., the sign of tearful eyes from the symptom of sadness). Radical separation of symptoms and signs is an epistemological impossibility, however, because as mentioned, the patient manifests himself through certain meaningful wholes, which typically emerge from a certain conjunction of the outer and the inner.

Here, the notion of Gestalt helps to express the wholeness of the clinical picture. A Gestalt is a salient *unity* or organization of phenomenal aspects. As is well known, the Gestalt cannot be reduced to a simple aggregate; the “whole is more than the sum of its parts.” This unity emerges from the relations between component features and is influenced by the whole (part–part-whole relations). A Gestalt may have *aspects*, of course, and these may be focused on in diagnosis or research; but one must remember that the aspects are interdependent in a mutually constitutive and implicative manner. The salience of, for example, an interpersonal encounter is *jointly constituted* by the patient’s experience, belief, and expression (“inner and outer”). “*What*” he says (content) is always molded by the “*how*” (form) of his way of thinking and experiencing. Further, a Gestalt instantiates a certain *generality of type* (e.g., this patient is typical of a category X), but this typicality is always modified, because it is necessarily embodied in a particular, concrete individual, thus deforming the ideal clarity and universality of the type [44]. We always perceive expression (sign) in the context of its temporal unfolding and in conjunction with the expressed contents (symptoms) and vice versa. This issue has been clarified in a classic article by anthropologist Clifford Geertz [19] who (borrowing from the philosopher Gilbert Ryle) describes the crucial difference between perceiving what may be the very same physical movement as a wink versus as a mere blink, depending on context and ascribed expression or intent.

It would be a mistake to think that the task of understanding the world requires only the discernment of identical elements across different individuals, together with measurements of quantity on specified dimensions. It is crucial as well to be concerned with forms of pattern recognition that involve *qualitative similarities*, whether of entire Gestalts or of aspects thereof. This is obviously the case whenever human expression is involved (as demonstrated by the entirety of the humanities disciplines—for example, history, literary, and cultural studies); but it is also the case in the natural sciences, perhaps most obviously in biology (discerning of structural and functional similarities is obviously crucial for evolutionary biology). Insistence on holism and Gestalt qualities is not therefore anti-scientific: it is possible both to compare Gestalts and to investigate their interdependent aspects in ways that allow for scientific generalizations. An illustrative example of the application of Gestalt analysis to psychiatry is the seminal work of Klaus Conrad on the beginning of schizophrenia [8].

The prevalent view or treatment of the psyche as a mere assemblage of the inner and the outer is reliant on the Cartesian dualisms of mind versus world and mind versus body that are now almost universally rejected in philosophy of mind and action. Contemporary philosophers of mind certainly recognize the experiential *asymmetry* between the first- and the third-person perspectives [48]; they also point, however, to the public or intersubjective dimensions of experience, perhaps most clearly manifest in emotion. In the case of emotions, the lived or subjective aspects cannot be separated either from the context in which they occur or from the associated bodily states, tendencies, and forms of expression with which they are associated—as both Wittgenstein and Merleau-Ponty have emphasized. “I could not imagine the malice and cruelty which I discern in my opponent’s looks separated from his gestures, speech and body,” writes Merleau-Ponty. “None of this takes place in some otherworldly realm”, in some shrine located beyond the body of the angry man [—] anger inhabits him and blossoms on the surface of his pale or purple cheeks, his blood-shot eyes … [45].

## Symptom and sign

A general account of consciousness and its phenomena, such as symptoms (experiences) and signs (expressions), can nowhere be found in the major contemporary textbooks of psychiatry in English (but see [70]. The prevailing but implicit assumption (evident in the psychometrics of research literature) seems to be that psychiatric “symptoms and signs” should (ideally) be treated as being close to third-person data: publicly accessible and involving mutually independent (and typically atomic) entities devoid of subtle or complex forms of meaning, and suitable for context-independent definition and measurement. The symptom/sign and its presumed, underlying causal substrate are assumed to exhibit the same ontological and descriptive nature: both are treated as spatio-temporally delimited entities, *thing*-*like* in nature. In this paradigm—adequate and fruitful in somatic *medicine*, where it originates—symptoms and signs have *no* intrinsic *sense or meaning*. Their role is to guide us toward underlying physiological causes. But the psychiatrist, confronting the “psychiatric object” [4, 42], finds himself in a radically different situation [32, 65]. She confronts not *a thing or body part* but a *person,* another embodied consciousness and its realm of meaning. And with very few exceptions, we do not, in fact, know the causal referents in any diagnostically relevant sense. What the patient manifests is not an isolated series of independent *referring symptoms/signs* but rather certain wholes of interpenetrating experiences, feelings, expressions, beliefs, and actions, all permeated by biographical detail. These aspects and these wholes are not constituted by a reference to underlying substrate (“extensionality”) but by their meaning (“intensionality”).

## The matrix of symptoms and signs: consciousness

Consciousness (mentality; subjectivity) is understood here as phenomenal manifestation of thoughts, feelings, perceptions, that is, broadly speaking, experiences. Consciousness is not lived as a spatial, three-dimensional object or as a *thing*, but more as a presence to itself and the world, as an inseparable dimension of our existence or life: Jaspers described “psyche” as “not […] an object with given qualities but as ‘*being in one’s own world*’, the integrating of an inner and outer world” ([32], p. 9, author’s italics). We apprehend the patient’s consciousness, his inner world (or first-person perspective), through and in his expressions and communications ([32], p. 20).

As countless philosophers and psychologists from the ancients to recent philosophers of analytic and phenomenological persuasions (e.g., [10, 63, 68, 76]) have noted, consciousness manifests itself as a becoming [32], a temporal flowing, and a “streaming” of intertwined experiences (including thoughts). This streaming is not amorphous but is organized into a *field of consciousness* (see [61] for an accessible account) which exhibits a certain structure, involving temporality, intentionality, embodiment, and self-awareness. In other words, consciousness does not consist of sharply separable, *substantial*, or thing-like components, exerting mechanical causality on each other. “Rather,” writes the phenomenologist Husserl “it is … a … network of interdependent *moments* (i.e., non-independent parts)…founded on intentional *intertwining*, *motivation* and mutual *implication*, in a way that has no analogue in the physical” [27], § 37. This peculiar nature of consciousness led Jaspers to deny any strict analogy between psychopathological description and the description in somatic medicine [32].

What, then, defines a given individual experience as a specific symptom, given that it is not pre-given as an autonomous, thing-like entity? On the phenomenological account, the symptom is *individuated* (becomes *this* or *that* symptom) along several dimensions, not only through its sheer content but also through its structure (form) and its meaning relations to previous, simultaneous, and succeeding experiences. Often, the symptom does not exist as a fully articulated “mental object” directly accessible to introspection or a preformed question, but rather as a pre-reflective, implicit content or as an altered framework/structure of consciousness. Frequently, it requires recollection. And in all these instances, articulation or individuation of a symptom requires a reflective, conceptualizing process that can be difficult to achieve.

Three examples may illustrate the issue of symptom determination as a meaningful whole inserted in a web of relations to other contents and forms of consciousness:

1. In our vignette (Table 1), the affirmative answer to the question on depressed mood in the structured interview is called into question in the conversational approach by bringing forth other experiences reported in the same thematic context,—and which do not belong to any conventional concept of depressed mood (e.g., “making an experiment”).
2. A smile, taken as such (in itself) cannot be predefined as silly. The silliness of a smile only emerges within the context of the flow of expressions relative to a particular discourse. The same applies to the bizarreness of a delusion [7] or to defining features of overvalued ideation or magical thinking.
3. Finally, consider the symptom of “audible thoughts” at the pre-psychotic and psychotic phases of schizophrenia. The phenomenon of audible thoughts is not defined by its presumed acoustic loudness or pitch. It should be suspected rather when there is a structural change in the field of awareness, namely, a disintegration of the unity of inner speech thinking into its components of *meaning* (content) and *expression* (signifier; sign). The patient seems to *listen to*, or *attend to his “spoken” thoughts* (or to thoughts expressed in writing or other visual form) in order to grasp *what* he is thinking. Normally, of course, we simply *know* what we think while thinking, without any help from signs, and without any temporal or experiential gap between the subject and his thought [14, 40].

As noted above, these issues do not arise in the typical structured interview. They are, however, crucial to the conduct of a phenomenologically oriented interview.

## The issue of language

Language is primarily directed toward the world. It seems obvious that language must have evolved to serve goal-directed cooperation between humans rather than for sophisticated description of mental states. Indeed there is a massive disproportion between the vocabulary at our disposal for dealing with the world or each other and that suited for introspection (and even this latter vocabulary is shot through by spatial, reifying terms). Mental terms are highly polysemic (the same term may have several different meanings), for example, the word “depression,” which for people without psychiatric training often means feeling in a poor mental condition [50]. When a patient says “I feel depressed, sad, or down,” such statement may, *if further explored*, be found to indicate a bewildering *variety* of experiences with varying affinities to the concept of depression: not only depressed mood but also, for instance, irritation, anger, loss of meaning, varieties of fatigue, ambivalence, ruminations of different kinds, hyper-reflectivity, thought pressure, psychic anxiety, varieties of depersonalization, and even voices with negative content, and so forth. In our vignette (Table 1), the affirmative answer to the question of lowered mood appeared to cover a whole array of mental states. Moreover, mood is not an *isolated mental object*, easily dissociated from its experiential context and identified in an act of introspection (i.e., converted to a reportable symptom). It is, so to say, a pre-given and pre-reflective manner of our experiencing [18, 69], something that, to the one who lives, is almost too immediate and encompassing to be recognized as such [24]. It therefore requires a careful interviewing effort to specify the salient profile of the presented distress. Taking a confirmatory or disconfirmatory answer at face value endangers the validity of the response.

This is one of the core issues confronting the psychiatric interview. First, as we argued already, the symptoms are not ready-for-use objects, ripe for the picking. Their final linguistic designation is an outcome of a conceptualizing process. An example might help us here: an abnormal experience of strange bodily sensations might be verbalized by the patient as, for example, a distressing “feeling, as if there are electric vibrations in my spine.” Here an anomaly in the field of awareness, an alien feeling or sensation, immediately mobilizes reflective attempts to conceptualize and describe (perhaps to regain a sense of control and intersubjective belonging [5]. This process is aided by *metaphorical* means, pre-eminently by metaphors involving space and energy that are linked to basic bodily sensory-motor modes [33, 39]. Here, the complaint becomes localized to an anatomical structure (the spine) that is usually phenomenologically mute. The description is therefore strange, yet still accessible to intersubjective understanding. The phrasing of “electric vibrations” gives us at least a glimpse of “what it is like” to have this symptom (which is a meaning-generative effect of the metaphor). Perhaps, a further concretization of this metaphor, away from its intersubjective anchoring, would make the statement close to a delusion. But in our example, the conditional “*as if* there are electric vibrations in my spine,” indicates that the patient has retained a grip on reality and maintains a reflective distance to his experience. He does not say, for example, “I *know* that there are electric vibrations in my spine,” in which case we might well consider him delusional.

## The interviewer: his role and activity

In the structured approach, the interviewer must faithfully asks in a pre-determined sequence, a series of closed pre-defined questions, corresponding to the diagnostic criteria. To maintain the purity of the quasi-experimental framework, it is crucial to minimize variance in the interviewer’s performance and, especially, to quash any potential tendency to inference and interpretation or any tendency for the patient to veer from the initial question.

By contrast the phenomenological approach is an eminently second person or I-thou situation in which interpersonal rapport is crucial. The interviewer is expected (and trained) to acquire a friendly and concerned attitude that is nevertheless neutral, non-invasive, and non-voyeuristic. It is extremely important to convey to the patient that even talking about the most bizarre experiences or fantasies is not beyond the psychiatrist’s professional competence and familiarity.

The latter point pertains to what is perhaps the most distinctively phenomenological aspect of the interview described here, an aspect that might be compared with the famous “phenomenological reduction” [27]. In ordinarily interaction with other people, we take for granted that we are all situated in a shared realm where certain things show up as “up there” or “real” or in various other ways such as “remembered,” “imagined,” and so on—in short, in accord with the “natural attitude” [27]. What a phenomenological interviewer attempts to do is to suspend the standard presuppositions of the shared, commonsense world, the unquestioned, commonsense background with its assumptions about time, space, causality, and self-identity, and about what does and does not exist as “real.” The point of this suspension is to make these assumptions (ordinarily overlooked) available to reflective awareness, and also to allow for the comprehension of lived worlds in which other ontological dimensions or presuppositions, for example, other forms of space, time, or causality might prevail.

The label “conversational interview” implies that the patient is encouraged to express himself freely and through reasonably uninterrupted narratives. Empirical research on witness interrogation has shown that a conversational approach, in which the witness is allowed to offer his own narrative, will enhance recollection and yield information that is more detailed and valid than does a series of closed questions [17, 30, 34]. In the course of the phenomenological interview, the narrative is the primary source of information, modified by context-fitting questions, requests for elaborations, details, and examples. Although the interviewer may occasionally propose an example, the patient’s reply is only considered valid if he or she is able to come up with an example from his own experience, or at least rephrase the example in his or her own words. Such a phenomenological approach serves to establish a rapport with the patient which extends beyond diagnosis to facilitate a therapeutic alliance.

Proponents of the structured interview are not, of course, unaware of the information variance ascribable to the patients’ cooperativeness. Nonetheless, the structured scheme seems to presuppose that the patient acts and responds as a rational consumer and motivated informant, involved in a mutually beneficial interaction. But patients arriving in a standard psychiatric facility (emergency, consultation, or a diagnostic unit) have very different backgrounds, trajectories (e.g., referrals from GPs, social/familial pressures, self-referrals, involuntary admission, etc.), and motivations to engage in a diagnostic dialog. Their abilities of self-description also vary; their experiences may verge at times on the ineffable. A significant number *dissimulate* in some fashion, typically by hiding aspects of psychopathology they consider shameful or strange. Sometimes, in fact, one only gets the targeted information by asking about something else or by not asking at all. As a rule, established rapport is decisive for the patient’s cooperation, especially in hostile or withdrawn patients. These common clinical constraints should proscribe any undue confidence in responses obtained, especially monosyllabic ones. All this is very well known, and has been discussed insightfully by sociologists, anthropologists, and other human or social scientists [38].

Under these circumstances, the psychiatric interviewer cannot be merely a passive receptacle of phenomenological data, but must actively participate in an interaction through which the symptoms unfold and are identified. The interviewer tries to represent the patient’s experiences and to disclose their typicality and distinctiveness, which means recognizing certain invariants or recurrent features. A crucial part of this process consists in a form of imaginative variation (in which most psychiatrists, though unknowingly, engage). This imaginative variation (akin to the “eidetic variation” or “eidetic reduction” of phenomenological investigation) is a conceptual operation whereby one attempts to imagine a phenomenon as being different from how it currently is in order to isolate which features or aspects might be essential—that is which features cannot be varied or deleted without preventing the phenomenon from being the *kind* of phenomenon that it is [55]. The prototypes acquired through clinical experience do not, then, derive simply from an averaging over time of a series of *mutually independent* sensory experiences. They are codetermined by a quest for *meaningful* interrelations between the observed phenomenal features, as when one looks for an “ideal type” [59].

## Limitations and conclusions

Our discussion of the *diagnostic interview* and its theoretical foundations should in principle be supplemented by an inquiry into the contemporary psychiatric *diagnostic systems*. Throughout the text, we tried to touch upon the ontological and the epistemological nature of the psychiatric object. However, an exhaustive address of the nosological issues is beyond our scope (see [36].

One possible objection to our insistence on the necessity of taking contextual, qualitative, and developmental aspects of the “object” of the psycho-diagnostic assessment into consideration might be that such emphasis is today obviated by the “top-down” operational classifications, with weak or absent hierarchy rules. The operational system, such as the DSM-IV, comprises classes, usually not mutually exclusive, and whose membership is based on a sufficient number of criteria. Thus, for example, a “major depression” is operationally (and nominalistically) defined, in disregard of any consideration on a phenomenological commonality (prototypicality) of depressive states, as a condition being *present* when a patient responds “yes” to a certain number of predefined questions. When the affirmative “yes answers” count as indicators of the diagnostic criteria, irrespective of the qualitative nature of the experiences (if explored) behind the answers (see Table 1), and irrespective of collateral information (e.g., years of increasing dysfunction, bizarre behaviors, and isolation), then, what was once called a “differential diagnosis” (i.e., a progressive narrowing down of diagnostic options over the course of the interview) is no longer necessary, relevant, or even possible (cf. the so-called “comorbidity debate”—[41].

However, there is an increasing recognition that the last 30 years’ operationalization and simplification of psychopathology has not resulted in visible gains in the validity of psychiatric categories [1, 20, 43]. Thus, for example, the concept of “evidence” in psychiatry is more complex than usually assumed [47].

McHugh advocates a return to a traditional “bottom-up” diagnostic interview, comprising: “(1) an extensive personal background history from birth to presentation, (2) a thorough description of the onset and progress of the presenting disorder, (3) a complete mental status examination as the patient first appeared to the psychiatrist […] (4) inquiry with ‘external informants’ who […] could provide contexts and continuities enriching what the distressed patient could report.” This proposal only makes sense if the diagnostic categories are not simply defined by “symptom counting” [43], that is, a sufficient number of context-free criteria but are anchored in some sort of descriptive commonality, prototype, or Gestalt [51]. In fact, it is now recognized that a change into a prototype-based approach to diagnostic classification is needed to improve the validity and utility of psychiatric categories in the forthcoming 11th edition of the International Classification of Diseases [74].

We realize that our focus on the modes of subjective experience, the notions of Gestalt/prototype and context, is likely to evoke a fear of looming unreliability and subjectivism (i.e., of a methodological regression, rekindling memories of the psychoanalytic domination of psychiatry). We are acutely aware of such dangers. However, the psychiatry’s perhaps most important task at hand is to regenerate psychopathology [29]. The focus on psychopathology is not incompatible with empirical rigor and reliability. We and others have empirically demonstrated that the semi-structured interviews focusing on subjective experience may, in fact, attain excellent interrater reliabilities, given sufficient clinical experience and training of the research psychiatrists or other clinicians who are involved [23, 72].

It should not be thought that the phenomenological enterprise in psychopathology is in any way opposed to the pursuit of scientific objectivity or to the project of neurobiological research, properly conceived [58]. The study of consciousness and even of self-experience has in fact become a central concern in contemporary neuroscience and cognitive science, as a spate of recent publications clearly shows, for example, [11, 15]. There is increasing interest in studying the neural correlates of such phenomena as emotion, the experience of selfhood, and the very fact of consciousness itself, but also in demonstrating the indispensability of the study of conscious life (understood as embodied and socially embedded) to the explanation of behavior in general [18, 71]. And it is widely recognized that this interdisciplinary enterprise—perhaps the central scientific challenge of the coming decades—will require paying close attention to the particular nature of subjective or mental life and its variations, and developing concepts and techniques adequate to this enterprise. To the extent that psychiatry ignores this challenge, it excludes itself from the forefront of scientific progress.

We need a methodological approach that is faithful to (mental or experiential) reality rather than an approach that implicitly distorts this reality in order to make it fit to its own prejudice. Faithfully to assess another person’s anomalies of experience, belief, expression, and behavior (the second-person perspective), adds certain specific demands to our clinical skills and analytic-conceptual knowledge, constituting psychiatry also as an *academic and scholarly* *endeavors,* while at the same providing solid foundations for achieving empirical objectivity.
